# A Case of Histoplasmosis with Central Nervous System Relapse after Itraconazole Therapy Needs Further Research

**DOI:** 10.7759/cureus.7064

**Published:** 2020-02-21

**Authors:** Prashanth Peddi, Tejo Challa, Sreenath Meegada, Madhavi Annakula, Evan Mar

**Affiliations:** 1 Internal Medicine, The University of Texas Health Science Center/Christus Good Shepherd Medical Center, Longview, USA; 2 Internal Medicine, Methodist Richardson Medical Center, Richardson, USA

**Keywords:** histoplasmosis, itraconazole, treatment failure, cns penetration, ring enhancing lesions

## Abstract

Central nervous system (CNS) histoplasmosis occurs in 5-20% of all cases and is most commonly seen in immunosuppressed patients who have acquired immunodeficiency syndrome (AIDS) or have received organ transplant. The prevalence of histoplasmosis in patients greater than 65 years old between the years of 1999-2008 in the state of Texas was about 2-3 cases per 100,000 patients year. Since 1990 with the discovery of Triazoles, itraconazole (ICZ) has become the standard initial and suppressive therapy in patients with mild-moderate histoplasmosis without CNS involvement. However, poor penetration of ICZ into the brain, in vitro fluconazole resistance and lack of controlled-trials pose challenge in the treatment of cerebral histoplasmosis.

## Introduction

Histoplasmosis is a fungal infection caused by Histoplasma capsulatum which is one of the common fungal respiratory infections, endemic in Africa, Latin America, Asia, central and southeastern states of the United States [1]. Most of the documented histoplasmosis infections are asymptomatic in immunocompetent individuals, and are self-resolving. Symptomatic cases of infections are seen in patients who are immunocompromised like acquired immunodeficiency syndrome (AIDS), transplant patients on immunosuppressants, patients with ventriculoperitoneal shunts, and patients on steroids [1]. Here we present a patient with history of adrenal histoplasmosis status post bilateral adrenalectomy presenting with central nervous system (CNS) histoplasmosis despite being on itraconazole (ICZ) therapy.

## Case presentation

**Figure 1:**
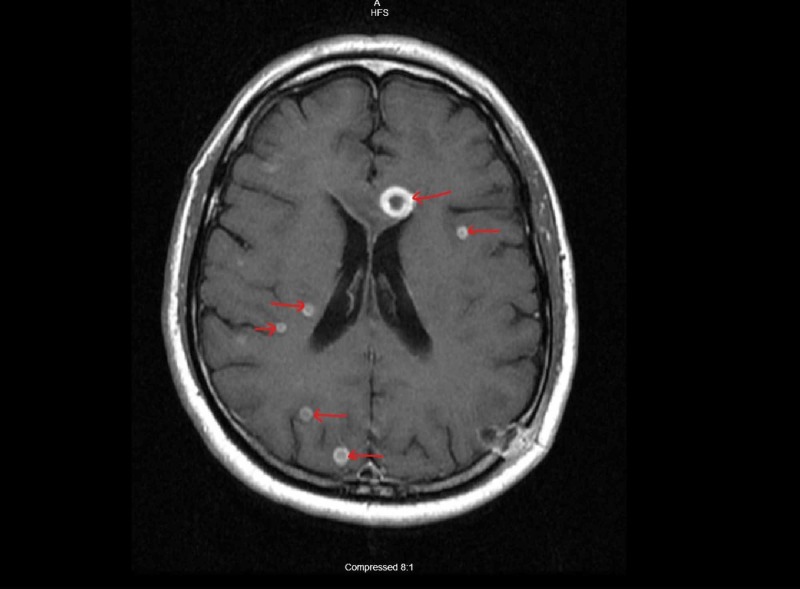
MRI brain with IV contrast showing ring enhancing lesions in cerebral cortex, one lesion close to ventricle (Arrows pointing)

**Figure 2:**
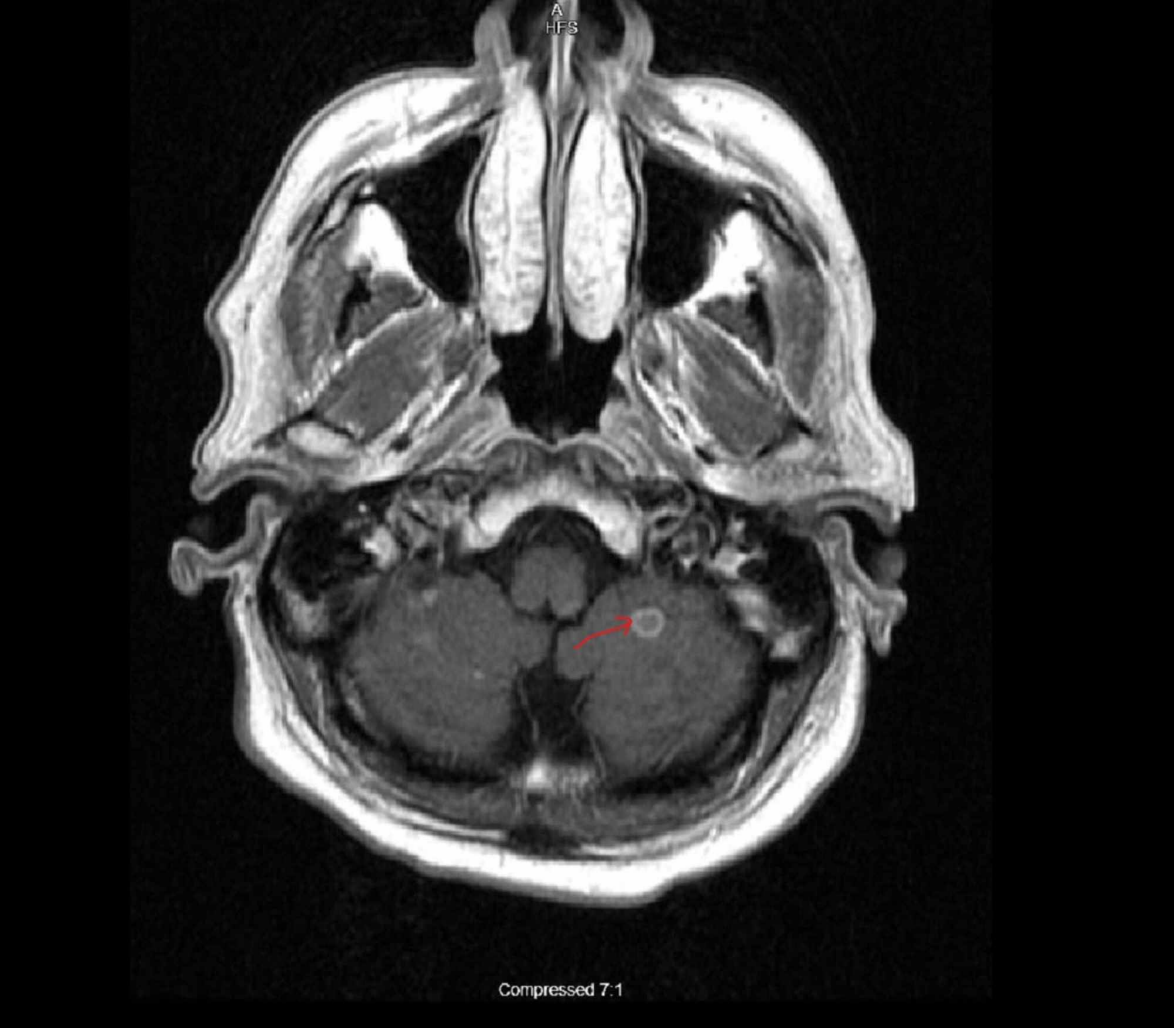
MRI brain showing ring enhancing lesion in cerebellar hemisphere (Arrow pointing)

**Figure 3:**
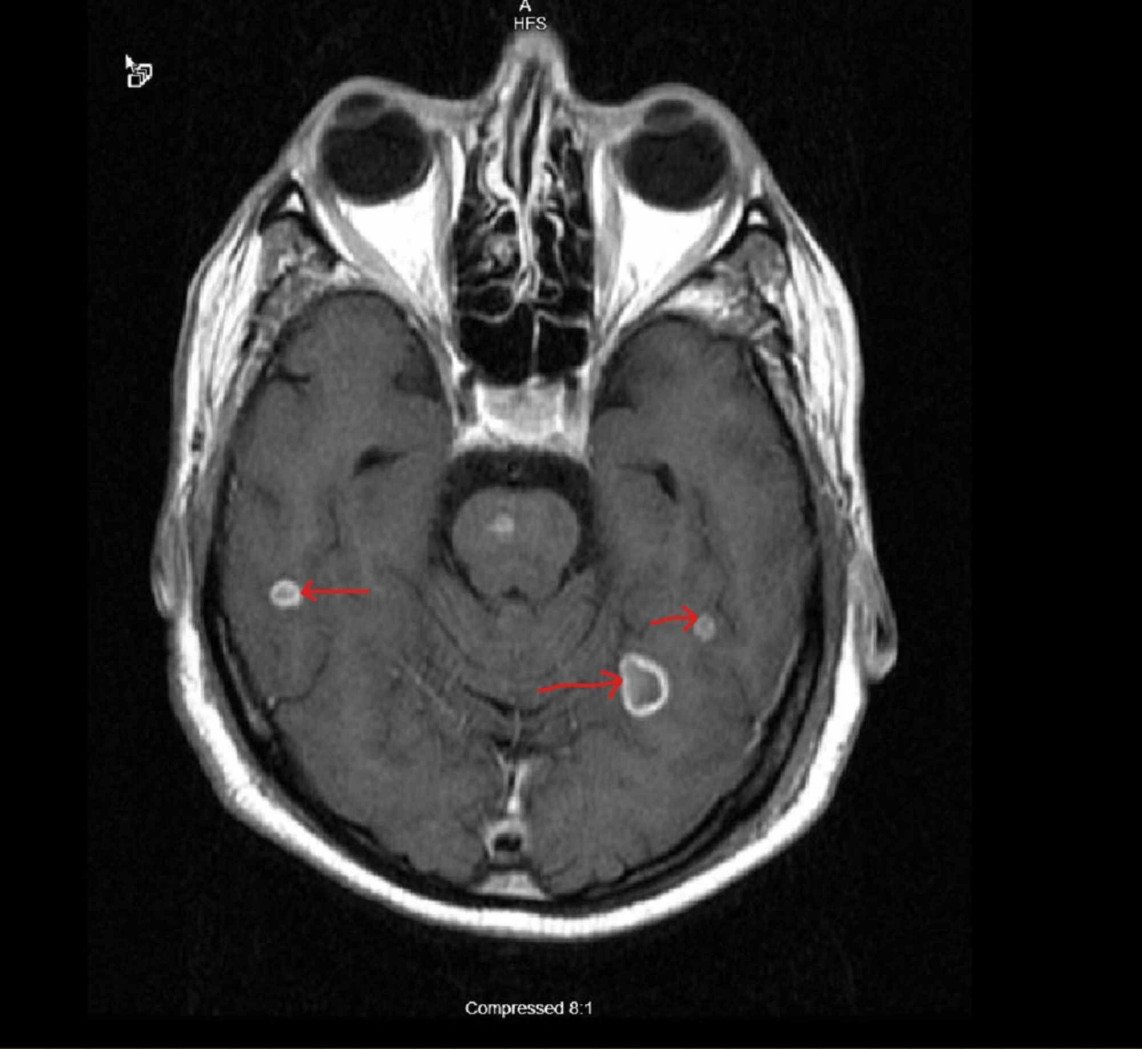
MRI brain different slice showing ring enhancing lesions in cerebral hemispheres (Arrows pointing)

A 56-year-old white male from Texas with history of adrenal histoplasmosis status post bilateral adrenalectomy who had completed two weeks of Amphotericin-B and one year of ICZ treatment presented to us with encephalopathy for one day and weight loss for two months. The patient’s wife was present and stated that the patient had a shuffled walk, poor memory, and when the wife attempted to walk the patient he would be falling to his left side repeatedly. The patient had immediate and remote memory intact. However, his recent memory was diminished and he was not capable of remembering the past 1.5 days. It was noticed that the patient had trouble with repetition in speech, and his fluency appeared to be slowed. In regards to cerebellar function, a patient is capable of performing rapid alternating movements but experiences some difficulty in performing finger-to-nose test and hesitation towards the end when attempting to touch a finger. The patient had initial computed tomography (CT) imaging performed which showed multiple areas of vasogenic edema involving the frontal lobes bilaterally and the left parietal lobe. Shortly after initial imaging, the patient had a magnetic resonance imaging (MRI) brain, which revealed ring enhancing lesions throughout the brainstem, cerebellum and cerebral hemispheres (Figures 1-3).

**Figure 4:**
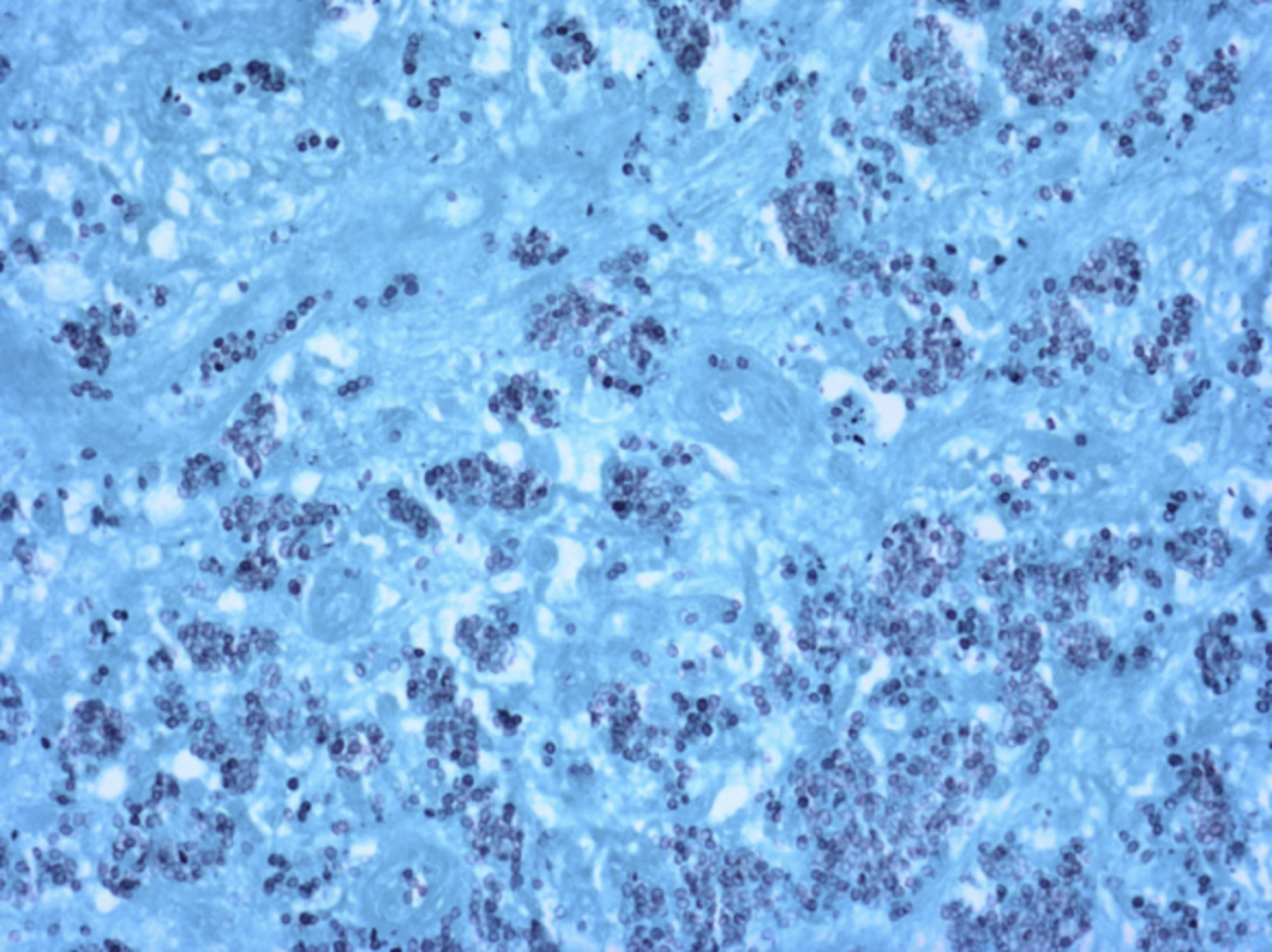
Patient’s open window brain biopsy with Gomori methenamine silver stain positive for histoplasma

The patient was started on IV Decadron whilst further workup was started and Infectious Disease was consulted. The patient was found to have an elevated erythrocyte sedimentation rate (ESR) of 48 mm/hr and C-reactive protein (CRP) of 19 mg/dL and urinary histoplasma antigen was positive. The patient was initially started on: Amphotericin B liposomal, Leucovorin, Pyrimethamine and Sulfadiazine. The patient also had negative toxoplasmosis and human immunodeficiency virus (HIV) workup. All treatments other than Amphotericin B liposomal were discontinued. An initial stereotactic brain biopsy and histoplasma polymerase chain reaction (PCR) were obtained, but after being examined both in house and after being sent to Washington University in St. Louis, MO, the sample was not able to decipher the diagnosis after 3-4 weeks. Tissue culture was positive for Histoplasma capsulatum per DNA probe. The patient experienced acute renal failure believed to be from Amphotericin B, so for a period of about two weeks before definitive diagnosis the patient was switched to IV fluconazole with resolution of his acute renal failure. Almost one month after the initial stereotactic biopsy, an open window brain biopsy of a granuloma occurred. Intraoperative pathology of the biopsy showed yeast consistent with histoplasmosis (Figure 4).

Subsequent tissue cultures confirmed the diagnosis. He was treated with Amphotericin-B for six weeks followed by long-term fluconazole. His neurological impairment improved considerably and he is now back to his baseline mental status.

## Discussion

The diagnosis of isolated CNS histoplasmosis can be quite challenging particularly in immunocompetent patients. However, a combination of high index of suspicion, clinical features, imaging studies and testing spinal fluid or urine for Histoplasma antigen might be useful. Identifying the histoplasma in the brain either by stereotactic or open biopsy remains gold standard for diagnosis of CNS histoplasma​ [1]​. Patients with CNS histoplasmosis can present with chronic meningitis, encephalitis, hydrocephalus, parenchymal lesions involving the brain and spinal cord resulting in stroke, seizures, confusion, memory impairment [1,2].​ Due to rarity of the CNS histoplasmosis and due to lack of randomized or comparative trials definitive treatment for CNS histoplasmosis in immunocompetent persons remains unclear. However, updated clinical practice guidelines issued in 2007 offered us guidance based on clinical experience and descriptive studies and provided two level III B recommendations which include 1) Liposomal amphotericin B (5.0 mg/kg daily for a total of 175 mg/kg given over 4-6 weeks) followed by itraconazole (200 mg two or three times daily) for at least one year and until resolution of CSF abnormalities, including ​Histoplasma ​antigen levels and 2) Blood levels of itraconazole (ICZ) should be obtained to ensure adequate drug exposure​ [3]. ​Checking the serum levels after initiation of therapy and monitoring clinical response by serial lumbar punctures, checking serum and urinary antigens is of paramount importance while patients undergoing proper treatment. With lack of treatment trials it is unclear what azole is preferred treatment for CNS histoplasmosis. ​Although ICZ has been found to be more active against H. capsulatum, it has poor penetration into the brain. P-glycoprotein-mediated efflux mechanism markedly limits the accumulation of itraconazole in brain tissue [4]​. This results in sanctuary sites in the CNS and relapse while ICZ is used as maintenance treatment after amp-B induction in disseminated histoplasmosis, which might be the cause of CNS relapse in our patient. Fluconazole achieves excellent concentration of drugs into the CSF as well as brain parenchyma​ [5]​. Review of the literature identified few reports where the use of fluconazole was highly successful despite its lower efficacy and high chance of developing resistance than itraconazole [6-8]​.​ ​Patients who do not tolerate itraconazole, or unable to achieve adequate blood levels with either preparation, or are receiving concomitant medications that lead to serious drug interactions with itraconazole, fluconazole may be alternative option like our patient who relapsed while on ICZ therapy which could be either patient non-compliance or intolerance due to drug.​ Newer azoles (posaconazole, voriconazole) also demonstrate in vitro activity against H. capsulatum similar to itraconazole; absence of in vivo data makes them less favorable​ [9]​. Until ​more research is done to establish the safety and efficacy of these new azole medications, these drugs are not encouraged to treat the CNS histoplasmosis.​ New drug delivery systems using nano-particle technology to achieve high concentrations of ICZ into brain tissue might be an option in the near future [10,11].

## Conclusions

Patients with disseminated histoplasmosis should be screened for CNS histoplasmosis as it may help to determine the type of treatment. Fluconazole has higher CSF and brain penetration. There are reported cases of successful treatment of CNS histoplasmosis with fluconazole. While it remains an alternative option for patients who do not tolerate itraconazole or fail to achieve target serum drug levels, more studies are needed to establish dosing, safety and efficacy. Newer azoles like posaconazole, voriconazole with in vitro activity against H. capsulatum and new drug delivery systems to achieve better CSF penetration show promise for treatment options in the future. Our case report highlights the ​need for further research in terms of standard of care for CNS histoplasmosis treatment.
